# Safe Production Strategies for Soil-Covered Cultivation of Morel in Heavy Metal-Contaminated Soils

**DOI:** 10.3390/jof9070765

**Published:** 2023-07-20

**Authors:** Xue Li, Tianhong Fu, Hongzhao Li, Bangxi Zhang, Wendi Li, Baige Zhang, Xiaomin Wang, Jie Wang, Qing Chen, Xuehan He, Hao Chen, Qinyu Zhang, Yujin Zhang, Rende Yang, Yutao Peng

**Affiliations:** 1 School of Pharmacy, Zunyi Medical University, Zunyi 563006, China; lee2521@foxmail.com (X.L.); tianhongyx@foxmail.com (T.F.); leewendi@foxmail.com (W.L.); zyj2003lj@163.com (Y.Z.); 2 Soil and Fertilizer Research Institute, Guizhou Academy of Agricultural Sciences, Guiyang 550006, China; xminwang22@126.com (X.W.); qinyuzhang22@126.com (Q.Z.); rendey@163.com (R.Y.); 3 Faculty of Food Science and Engineering, Foshan University, Foshan 258000, China; hongzhao0515@outlook.com; 4 Key Laboratory for New Technology Research of Vegetable, Vegetable Research Institute, Guangdong Academy of Agricultural Sciences, Guangzhou 510640, China; plantgroup@126.com; 5 Qiandongnan Academy of Agricultural Sciences, Kaili 556000, China; jiewang52@21cn.com; 6 College of Resources and Environmental Sciences, China Agricultural University, Beijing 100193, China; qchen@cau.edu.cn; 7 School of Pharmaceutical Sciences, Sun Yat-sen University, Shenzhen 518107, China; hexh26@mail.sysu.edu.cn; 8 School of Agriculture, Sun Yat-sen University, Shenzhen 518107, China; chenh626@mail.sysu.edu.cn

**Keywords:** heavy metals, mitigation strategies, morel, mushroom, soil remediation

## Abstract

Morel is a popular edible mushroom with considerable medicinal and economic value which has garnered global popularity. However, the increasing heavy metal (HM) pollution in the soil presents a significant challenge to morels cultivation. Given the susceptibility of morels to HM accumulation, the quality and output of morels are at risk, posing a serious food safety concern that hinders the development of the morel industry. Nonetheless, research on the mechanism of HM enrichment and mitigation strategies in morel remains scarce. The morel, being cultivated in soil, shows a positive correlation between HM content in its fruiting body and the HM content in the soil. Therefore, soil remediation emerges as the most practical and effective approach to tackle HM pollution. Compared to physical and chemical remediation, bioremediation is a low-cost and eco-friendly approach that poses minimal threats to soil composition and structure. HMs easily enriched during morels cultivation were examined, including Cd, Cu, Hg, and Pb, and we assessed soil passivation technology, microbial remediation, strain screening and cultivation, and agronomic measures as potential approaches for HM pollution prevention. The current review underscores the importance of establishing a comprehensive system for preventing HM pollution in morels.

## 1. Introduction

Morel (*Morchella* spp., *Pezizales*, *Ascomycota*) is a macro fungus with a distinctive fruiting body full of stomata [1]. Known for its high nutritional and medicinal value, morel is one of the most valuable medicinal mushrooms worldwide [2,3]. It is a rich source of high-quality protein, various amino acids, unsaturated fatty acids, polysaccharides, and multiple mineral elements [4,5]. Morel has been found to have important effects on the kidneys and liver, as well as antibacterial, anti-inflammatory, antioxidant, and anti-diabetic properties [6,7,8,9,10]. In addition to its medicinal value, morel has significant economic importance due to its worldwide distribution, with prominent populations found in China, the United States, France, Spain, and Turkey, as well as in Peru, Ecuador, Venezuela, and India [4,11,12,13].

Morel was once a soil-saprotrophic ascomycete mushroom that can now be cultivated routinely in farmland soils [14]. The artificial cultivation of morels started in the United States and was cultivated on a large scale in China. In 2021, the global export value of morel reached USD 9.6 billion, with China being the largest exporter. The artificial cultivation area of morel in China has grown from less than 10,000 acres to more than 200,000 acres, with cultivation areas in more than 20 provinces across the country [15]. Cultivation morel is an attractive and profitable industry due to its low cultivation costs, high production value, and better economic benefits compared to other edible mushrooms that require costly and complex cultivation systems. In addition, morels have unique and delicate flavors and textures, which makes them a popular ingredient in high-end cuisine worldwide.

Morels always use the soil as the substrate for forming fruiting bodies. Therefore, the entire lifecycle of morel, including mycelial growth, primordia formation, and fruiting body development, takes place within the soil environment. Meanwhile, morels have a positive impact on soil fertility and soil microbial community structure. Soil microorganisms such as *Arthrobacter, Bradyhizobium*, *Devosia*, *Pseudarthrobacter*, *Pseudolabrys*, and *Nitrospira* that grow around morels have nitrogen fixation and nitrification abilities [16]. In addition, morels improve soil microbial community structure, which contributes to soil remediation and improves soil use efficiency.

The proliferation of human activities and accelerated industrialization has engendered the proliferation of heavy metal (HM) contamination in soil, as corroborated by Adnan, et al. [17]. The contamination of soil with HMs is a potential menace to the production and quality of morels, food safety, and human health, as HMs are not biodegradable and persistently accumulate in the soil [18]. The 2014 National Soil Pollution Status Survey Bulletin posits that the total exceedance rate of soil in China was 16.1%, with arable, forest, and grassland soil sites having exceedance rates of 19.4%, 10.0%, and 10.4%, respectively. Cadmium (Cd), arsenic (As), and copper (Cu) have been identified as the most widespread HM contaminants in the region. The Cd limit of morel was 0.6 mg kg^−1^, and As limit was 0.5 mg kg^−1^ according to GB 2762-2022 in China [19]. The escalation of soil HM contamination has led to HM contamination of morels in some areas, as exemplified by HM content testing of 59 batches of morels in Qianxinan, Guizhou, where Cd exceeded the limit by 33.9% and chromium by 1.7% [20]. Similarly, morels collected from apple orchards contaminated with lead (Pb) arsenate in the eastern United States exceeded the standard for both Pb and As [21]. The HM content of morel is higher compared to some plants and other edible mushrooms [22,23,24,25]. Morel is highly susceptible to soil HM content, and the HM content in the fruiting bodies is positively correlated with the HM content in the soil [26]. The uptake and biosorption of HMs in morel lead to their enrichment in various forms, such as compartmentalization, exclusion, complexity rendering, and so on [25,27,28].

**Figure 1:**
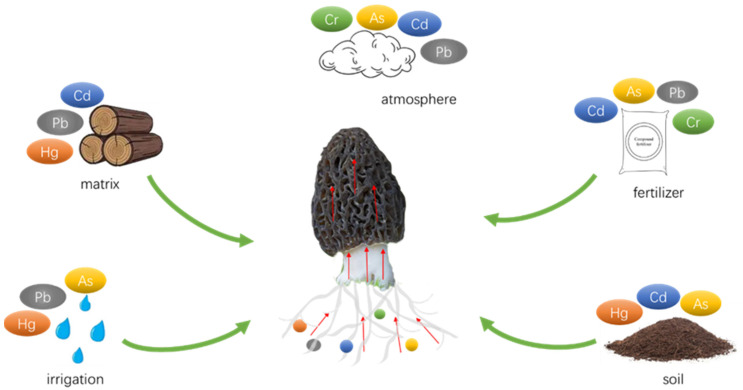
Sources of heavy metals in morel.

In the pursuit of reducing HM accumulation in mushrooms, many research studies have focused on optimizing cultivation substrates and methods, as well as the incorporation of beneficial metal elements. For example, Weng, et al. [29] utilized forage as an alternative to wood chips in the cultivation process, which resulted in a significant decrease in HM levels including Cd, Pb, and Cr, while also improving the overall quality of the mushrooms. Jiang, et al. [30] introduced selenium and lanthanum complex reagents (Na_2_SeO_3_ 87.619 mg kg^−1^, LaCl_3_ 70.670 mg kg^−1^) to the growth medium of *A. brasiliensis*, leading to a reduction of HMs such as Pb, Cd, Hg, and As in the fruiting bodies by 11.58%, 55.24%, 46.43%, and 52.38%, respectively. Some HM binding sites on the cell wall of morel are non-specific, and Cd^2+^, Zn^2+^, and Mg^2+^ are usually taken up by the same transporter [31,32]. Se, Zn, or Mg can form complexes with Cd in soil, which exhibit relatively stable structures and ultimately reduce substrate uptake and cellular uptake of Cd [33,34]. However, the sources of HMs in morels also include soil, atmosphere, and water (Figure 1). The most direct and effective solution to reduce HM accumulation in morels is to minimize soil contamination. Various techniques for soil HM remediation, including physical, chemical, and bioremediation approaches, have been well-established and widely implemented.

**Figure 2:**
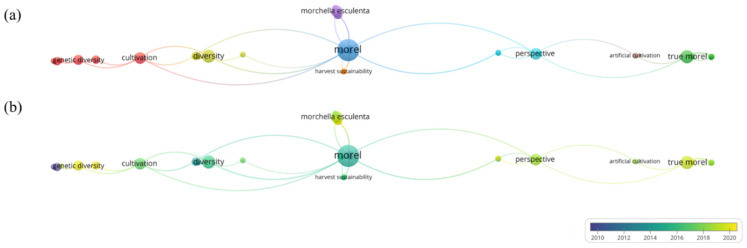
Bibliometric analysis of the theme. (**a**) Topic distribution. The map shows three clusters. Orange clusters represent harvest sustainability. Red and brown clusters involve artificial cultivation, growth characteristics, and gene diversity. Blue and green clusters represent the variety and perspective of morel. (**b**) Trend topic network diagram based on keywords used from January 2000 to December 2020. Indicators show the current publication from blue to green. Review of morel recently published. The size of the circle represents the frequency of keywords. The distance between the two circles indicates their correlation.

Up to now, there are 59 reviews of morels on Google Scholar, including 54 articles and 5 books, mainly covering the classification of morels, the progress of artificial cultivation, fungal diseases, genetics and systematics, chemical composition, and pharmacological effects, but there are no reports on HM control strategies of morels, as shown in Figure 2. However, there are 237 articles related to “morel” and “HMs” in PubMed, and HM pollution control technology in morels has received wide interest. Therefore, the review summarizes the mechanisms of HMs that are easily enriched during the cultivation of morels and evaluates soil passivation technology, microbial remediation, strain screening and cultivation, and agronomic measures as potential ways to prevent HM pollution. The importance of establishing a comprehensive system to prevent HM pollution in morel was emphasized.

## 2. Morel Varieties and Cultivation Methods

### 2.1. Main Morel Varieties

In Europe, Asia, and North America, a diverse range of morel species can be found, with 34, 32, and 21 species, respectively [35]. China, particularly Yunnan, Sichuan, Guizhou, Chongqing, and Tibet, is a center of species diversity of morels, with several species widely distributed in the region [36,37,38,39,40]. Notable species include *M. esculenta*, *M. crassipes*, *M. spongiola*, *M. conica*, *M. elata*, and other species [41,42]. However, due to seasonal and quantity limitations, it is challenging to meet the demand for wild morels, which fruit primarily in the spring season, with a few species fruit in summer or autumn [43]. Morels have been observed to emerge in the autumn in Israel and the Southwest Himalayas; the species and timing of their emergence are erratic and may be influenced by local environmental factors such as precipitation, temperature, or the life cycle of morels [44,45,46]. Consequently, the artificial cultivation of morels has become a popular research topic. Artificially grown morels, which are similar to wild morels in nutritional value but contain lower levels of HMs, are safer and more cost-effective [47]. Currently, there are nine successfully domesticated cultivars of morels, including Esculenta Clade (*M. conica*, *M. angusticeps*, *M. importuna*, *M. sextelata*, *M. eximia*), Esculenta Clade (*M. cassipes*, *M. esculenta*, *M. deliciosa*), and Rufobrunnea Clade (*M. rufobrunnea*) [48]. Of these, only *M. importuna*, *M. sextelata*, *M. eximia*, and *M. rufobrunnea* have been cultivated on a large scale for commercial purposes, with *M. importuna* being the most widely cultivated species globally and accounting for over 95% of the total cultivated area [49]. There may be differences in the accumulation characteristics of HMs elements in the same variety of morel strains (Table 1) [50]. Therefore, directional selection of HM-resistant strains that meet the breeding objectives can effectively control the accumulation of HMs in morels.

### 2.2. Cultivation Methods

Since 2012, the cultivation of morels has been making steady progress, with a new emphasis on field and forest cultivation through exogenous nutrient bag technology [48]. Field cultivation is a smart and cost-effective option, but it comes with its fair share of challenges, as it can be vulnerable to the whims of nature—think sudden temperature changes, drought, and gale-force winds. However, understory planting in evergreen forests with crown densities above 80%, such as fir forests, viburnum forests, and citrus forests, can mitigate these risks. More importantly, we should stay away from industrially developed farmland soil and avoid surrounding mines. Reducing HM pollution sources is one of the effective means to reduce the content of HMs in morels.

**Figure 3:**
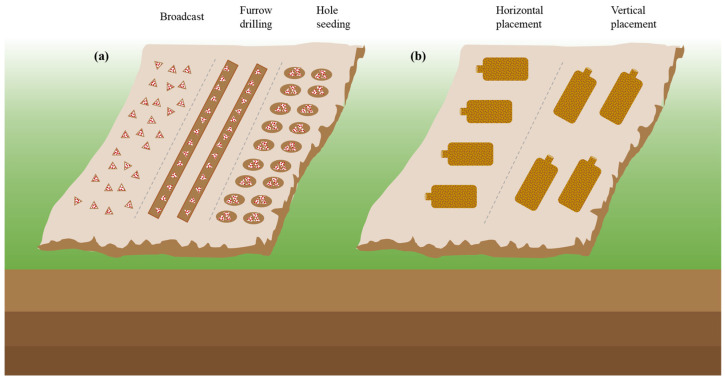
(**a**) The sowing methods and (**b**) nutrition bag placement.

Sowing methods and soil conditions are also crucial for successful cultivation. Common sowing methods include furrow, spreading, and hole sowing, as shown in Figure 3, and nutrient bags are typically spaced 20–30 cm apart after 7–15 days of sowing [58,59,60]. In Chongqing, Li, et al. [61] found that the content of HMs in edible fungi collected in different seasons was also different. The content of HMs in *Volvariella volvacea*, *Pleurotus ostreatus*, *Lentinula edodes*, and *Flammulina velutipes* was high in winter and lowest in spring. But the best time to sow a wide range of *M. importuna* is in October, with a harvest period from February to March, because the fungi are known for their love of water and low-temperature tolerance, with an optimal growth temperature of 25 °C [62,63]. Fortunately, with the development of science and technology, intelligent mushroom houses can make the cultivation of morel without a time limit. Traditional field cultivation requires a soft, flat terrain position with favorable water and clay or sandy soil mixed with humus soil, which provides the necessary nutrients and space for morel mycelial growth [35,48].

Industrial cultivation of morels can be achieved through indoor or outdoor cultivation, each with its respective advantages and disadvantages [64]. The HM content of mushrooms grown under different cultivation methods is also different. For example, soilless cultivation of *Grifola frondosa* can effectively block the enrichment of Pb by its fungi [65]. The growth of *Cordyceps militaris* fruit bodies exhibited a proportional inhibition in response to the presence of Pb, Hg, and Cd in the growth medium, displaying a dose-dependent relationship [66]. For morels, the Cd content under different cultivation modes can be ordered from high to low as a soil covering cultivation > layer frame cultivation > oblique insertion cultivation [67]. At the same time, we should avoid the occurrence of HMs in the cultivation matrix. As the initial site of mycelium development, mushrooms showed a higher tendency to absorb cadmium from the matrix [68]. Equally important is the raw material of the nutrition bag, which must ensure that there is no HM pollution source; otherwise, it will directly lead to the pollution of morel.

## 3. Mechanisms of HMs Uptake by Morels

Several studies have highlighted the presence of HM enrichment in morel, including Cd, Cr, As, Pb, and Hg [69,70,71]. For instance, Mohammad, et al. [24] observed bioaccumulation factors (BF) of HMs in *M. esculenta* to be Cd (0.84), Cu (0.8), Co (0.69), Pb (0.61), Ni (0.6), Mn (0.51), and Cr (0.3). Despite these findings, the intricate process of HM uptake by morel remains unknown. This may be attributed to the fact that morel requires specific elements to promote its growth or initiate a series of self-defense mechanisms to mitigate the harm caused by HMs [70]. For instance, Fe has been found to promote the growth of *M. conica* mycelium and the formation of fruiting bodies, while Zn application increases the content of amino acids in fruiting bodies. Additionally, HMs may exist in various forms within the cell. In a recent study, Xiong, et al. [72] employed HPLC-ICP-MS to examine various mercury forms in rambutan, indicating that morel’s uptake of Hg involves four forms: methylmercury, ethylmercury, inorganic mercury, and phenylmercury. Nevertheless, no comprehensive research has been conducted to explicate the underlying mechanisms of HM enrichment in this fungus. Existing studies suggest that morel’s HM enrichment involves two processes: (i) cellular active uptake, which is dependent on cell metabolism, and (ii) biosorption, which is not dependent on cellular metabolism and includes extracellular accumulation, cell surface adsorption, and intracellular accumulation. These findings are consistent with previous observations on HM enrichment by mushrooms.

### 3.1. Cellular Active Absorption

**Figure 4:**
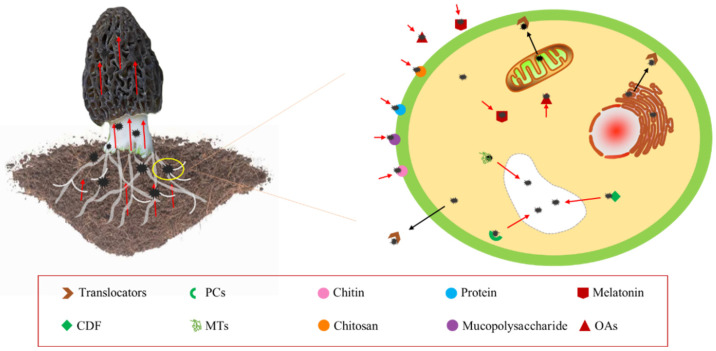
Enrichment mechanism of heavy metals by morel.

Morels have various protection mechanisms against HMs stress like compartmentalization, exclusion, complexity rendering, and the synthesis of binding proteins, including phytochelatins (PCs), metallothioneins (MTs), Cd-binding peptides (Cd-BPs), cysteines (Cys), and histidines (HIs) [73]. They play crucial roles in the signaling, uptake, detoxification, and accumulation of metal [74]. HMs that enter the cell can be sequestered in vesicles through the action of various PCs, MTs, glutathione (GSH), or specific metal-binding ligands, as shown in Figure 4 [75,76,77]. This mechanism reduces HM toxicity within the cell, which is a major contributor to HM enrichment in the mycelium. PCs and MTs are classes of Cys-rich proteins that bind HMs with high affinity and form complexes segregated into vesicles in plants and fungi [78,79,80].

Intracellular signaling molecules, such as hydrogen sulfide (H_2_S), also play a role in HM uptake. H_2_S has been found to activate Cd^2+^/H^+^ reverse transporter proteins on the vesicle membrane, resulting in increased Cd sequestration in the vesicle [81]. Moreover, cation diffusion facilitators (CDFs) have been identified as a recently discovered family of proteins responsible for HM ion transport. CDFs are capable of transporting a single cation or multiple divalent cations in many cases [82,83]. In the yeast *S. cerevisiae*, CDFs transport metals from the cytoplasm to the vesicles [84].

### 3.2. Biosorption

The uptake of HMs by morel involves physicochemical interactions of functional groups present on the cell surface, which allow for electrostatic adsorption, ion exchange, precipitation, and complexation without requiring cell metabolism, as illustrated in Figure 4 [85,86,87]. In Cd stress, 50% of Cd is bound to the cell wall, 30% remains in the cytoplasm, and 20% is translocated to the vesicles [88]. The cell wall’s major components, such as titin, chitosan, mucopolysaccharides, and proteins, form chelates with HMs by providing adsorption, ion exchange, and covalent binding sites, including carboxyl, hydroxyl, sulfhydryl, amino, and phosphate groups [89]. HMs are immobilized on the cell surface through ion exchange, complexation, and precipitation [90,91,92]. Chitin, a biopolymer found in the shells of marine crustaceans and fungal cell walls, has been used to extract HMs such as Cu, Zn, Cd, Ni, and Pb from water, owing to its ability to bind HMs through amino and hydroxyl groups [93,94,95,96]. The chitin content of morel has been reported to be around 16% [97].

In addition to these mechanisms, Liu, et al. [98] observed that a cysteine-rich hydrophobic protein on the morel cell wall can form chelates with HM ions due to the presence of sulfhydryl groups in cysteine. Under HM stress, the cell wall secretes melatonin and organic acids that bind to HMs, thereby reducing stress [99]. Laccase, widely present in morel cells, is an enzyme that catalyzes melanin synthesis [100]. Wang, et al. [101] found that laccase activity presented downtrends as the concentration of Cd increased, which might be the promotion of more laccase synthesis of melatonin under Cd stress. Higher laccase levels catalyze the synthesis of melanin, which deposits HMs outside the cells.

## 4. HM Mitigation Strategies

HM-contaminated soil usually includes two methods: source and process blocking. The source-blocking methods mainly include strain screening and breeding (e.g., radiation breeding, transgenic breeding, and hybrid breeding) (Table 2). Process blocking techniques include physical remediation (such as soil replacement, soil washing, vitrification, electrokinetics remediation, and thermal treatment), chemical remediation (including immobilization, extraction, and chemical leaching), and bioremediation (such as phytostabilization, phytoextraction, phytovolatilization, microbial remediation, microbial-assisted phytoremediation, and animal remediation) [102,103,104,105]. Among these techniques, bioremediation and soil stabilizers are low-cost and environmentally friendly, with minimal damage to soil components and structure. Microbial remediation offers an additional advantage in that it can utilize the complex and diverse microbial communities present in the growing environment of morels. These communities form a harmonious symbiotic relationship with the mushrooms. Similarly, soil amendments like biochar do not cause damage to the soil structure and can even provide more growth space for both the morels and microbes.

### 4.1. Screening and Cultivation of Suitable Strains

During domestication and cultivation of wild morels, the screening and breeding of low HM accumulation varieties represent an effective strategy for reducing HM content in mushrooms. Notably, HM accumulation characteristics may differ even among edible mushroom strains of the same species. Yu, et al. [128] used transcriptomic analysis to investigate two genotypes of *Lentinula edodes* with differing Cd accumulation capacities and found that the high Cd accumulation type Le4625 had approximately three times more Cd than the low Cd accumulation type Le4606. Additionally, transcriptome and expression profiling of the molecular response of *M. spongiola* in Cd toxicity revealed the major detoxification pathways under Cd stress, including MAPK signaling, oxidative phosphorylation, pyruvate metabolism, and propanoate metabolism, offering a new pathway and possibility for bioremediation in Cd stress [143]. Breeding low HM accumulation varieties is a key research direction, with common mushroom breeding methods, including cross-breeding, radiation and chemical mutagenesis breeding, and transgenic breeding. Cross-breeding, which combines the positive traits of the parents, is the most widely used approach and can result in new mushroom varieties with higher yields, better nutrition, and greater resistance to viruses and HMs [144,145,146,147]. Radiation mutagenesis breeding is also commonly used in the breeding of edible mushrooms. For example, Liu, et al. [130] used Co^60^-γ-irradiation radiation to mutate a strain of *Agaricus brasiliensis*, resulting in increased yield and amino acid content, as well as reduced accumulation of As, Pb, and Cd in the substrates. As the morel genome continues to be sequenced, genes related to HM transport are being identified, and transgenic breeding tools will become increasingly relevant in mushroom breeding [148]. Chen, et al. [149] identified the Cd stress response gene ATX1 in Oryza sativa, and found that ATX1-silenced transformants showed enhanced Cd resistance, while ATX1 overexpressed transformants showed reduced Cd resistance. Thus, genetically modifying relevant genes (gene silencing, gene knockout, and gene overexpression) is a promising approach to obtaining HM-tolerant strains, although making these genes heritable remains a challenge [150]. Recently, a study on Agrobacterium-mediated genetic transformation successfully transformed the hygromycin resistance gene in *M. importuna*, providing a reference for the genetic system of *M. lamblia*. In addition, liposome transformation and electroporation represent novel approaches to make genes heritable in *M. importuna*, which warrants further investigation.

### 4.2. Agronomic Measures

Agronomic practices such as fertilization, intercropping, and water management have been employed to reduce HM content in morel. The application of standardized management patterns, including the use of non-polluting watering water and standard compound fertilizers, has been shown to effectively reduce HM content in morel [141]. Full-fertility flood irrigation has also been demonstrated to be more effective than wet irrigation and intermittent irrigation in reducing the biological effectiveness of Cd in Cd-contaminated soils. Some beneficial metal elements in fertilizer, including Zn [151], Fe [152,153], Se [154], Si [154], Ca [155], K [156], and Ce [157,158], play crucial roles in crop growth. The application of fertilizers with beneficial metal elements can effectively reduce the enrichment of HM elements in morels by antagonizing HM ions on the cell wall, as HM sites on the cell wall of morels are not specific [31,32]. For example, Yu, et al. [159] demonstrated that P fertilizer significantly increased soil pH and available P while decreasing soil available HM concentrations. Additionally, Zhou, et al. [160] effectively passivated Cu, Zn, and As by adding 3% Fe_2_(SO_4_)_3_ to pig manure, which led to a decrease of 82.35% and 80.00% in available Cd and Pb concentrations, respectively [161]. Moreover, foliar inhibitors containing Si/Se were found to reduce the available HMs and inhibit the absorption of Cd by plants in red soil [162].

The mycorrhizal symbiosis between morel and various plants, including yellow pine, poplar, spruce, maize, cheatgrass, and peach trees in the pine family, has been observed [139,140,163,164]. Interestingly, poplar, willow, maize, Indian mustard, sunflower, and vetiver are highly tolerant to HMs [165,166]. Vetiver has been used for revegetation in Pb and Zn mines, and some species of vetiver, such as *M. rufobrunnea* and *M. esculenta*, exhibit mycorrhizal symbiosis [167]. Song, et al. [140] used a Peach-vetiver intercropping mode to promote both. Therefore, the use of a plant-morel rotation/intercropping mechanism may be a promising strategy to reduce HM content in morel, but further research is required. In conclusion, the development of fertilizers that are both nutritious and have an HM-blocking effect is a promising area for future research.

### 4.3. Microbial Remediation

The soil microbiome undergoes a rich and complex community shift during the growth of morels. Benucci, et al. [168] identified 169 microbial communities associated with morels cultivated in greenhouses. Longley, et al. [169] further established that the potential microbial community structure in different strains of morel mushroom substrates is variable, and the functional roles of bacteria and fungi in these communities vary widely. For instance, *Pseudomonas putida* has been demonstrated to facilitate the evolution of morels from mycelium to nucleus [170], while *Pseudomonas* has also been shown to enhance the production and protoplast formation of *Agaricus bisporus* [171].

Recent studies have shown that bacteria and fungi can be employed for the remediation of HM contamination in soil (Table 3). *Bacillus thuringiensis* could sorb Cd by 97.67%, while *Bacillus laterosporus* was effective in remediating Cd and Cr [172,173]. Similarly, Khan, et al. [122] established that *A. fumigatus* and *A. flavus* could remove Pb with efficiencies of 99.20% and 99.30%, respectively. Meanwhile, nano-fungal chitosan was utilized to remediate Pb and Cu, achieving over 90% reduction in their levels when applied at 0.5% concentration [174]. Interestingly, different microorganisms respond differently to various HMs, and the most suitable microorganisms for inoculation can be selected based on the actual situation [175].

### 4.4. Soil Passivation Technologies

The application of soil amendments is an effective means of reducing HM contamination in light to moderately contaminated soils and mitigating the uptake and enrichment of HMs by organisms such as the fungus in the sheep maw. Some alkaline modifiers such as gypsum, lime, and plant ash are often used as HM passivation in the cultivation of morels. Morel will secrete some acidic substances during the growth process, which will reduce the pH and lead to the growth of pathogenic bacteria [191]. The application of amendments, such as lime, serves multiple purposes in soil remediation. It not only helps in pH adjustment and sterilization but also facilitates the formation of precipitates by binding OH^−^ with HMs, effectively immobilizing them in the soil [192]. However, excessive application of lime can lead to soil compaction, which reduces soil fertility and increases the risk of HM ion uptake by morels. For instance, Ca ions can activate the soil Cd and Pb ions through cation exchange processes. Therefore, other potential HM soil amendments may be suitable for morels, such as biochar (BC) [106,107], clay minerals [108], magnetic nanosorbents [109], natural zeolites [110], silica additives [111], montmorillonite-based amendments [112], and bone char [113], as shown in Figure 4. It is noteworthy that HM soil amendments suitable for morel should meet three conditions: (i) no damage to the mycelium, (ii) certain alkalinity, and (iii) a porous structure to provide space for mycelial growth or be rich in beneficial elements such as C, N, K, and Mg. BC is a promising passivation material due to its eco-friendliness and wide availability. Agricultural and municipal wastes such as rice straw, coconut shells, and animal manure serve as raw materials for BC [193,194,195]. Modified BC can be used to remediate various HMs such as Cd, Pb, Cu, and As [196,197,198,199].

Composting is an effective approach to restoring organic matter and blocking the uptake of HMs by morel [200]. The bioorganic fertilizer generated during composting alters the organic material’s surface structure and mechanochemical functional groups, enhancing its sorption properties [201]. The composting process also generates humic acids that bind and passivate HMs, thus reducing their toxicity [202,203]. Various edible mushrooms such as *Agaricus blazei*, *Pleurotus ostreatus*, *Lentinus edodes*, and *Agaricus bisporus* have been used as composting substrates, with more than 70% HM adsorption efficiency demonstrated [115,116,117]. The addition of earthworms and poultry manure to the composting process enhances its HM adsorption efficiency. Moreover, the use of edible mushroom waste substrate as composting material offers a promising approach to waste recycling. Local soil HM contamination conditions can guide the selection of appropriate soil amendments.

## 5. Conclusions and Prospects

China has emerged as a pivotal player in the edible and medicinal mushroom industry, boasting substantial production volumes, expansive export capabilities, and notable competitive advantages both domestically and internationally. Of particular interest and value are morels due to their high edible, medicinal, and economic worth. However, the development of the morel industry is facing a notable challenge in the form of HM pollution and exceedance. HMs are harmful to both morels and humans, with soil contamination reducing growth and yield. When exposed to HMs, morel cells generate oxidative stress responses and protective mechanisms, resulting in HM enrichment. It is thus imperative to minimize HM contamination in morels. Various strategies are employed to mitigate HM toxicity, including the cultivation of low HM accumulating morel strains, the use of microbial inoculants, and the application of soil passivation. The soil passivation, in particular, offers a potential source control solution that could be the most effective way to curb the enrichment of HMs by morels. In addition, microbial inoculants represent a promising strategy for the remediation of soil HM pollution and have already been productized. There is also a growing field of research on genomics, transcriptomics, and metabolomics related to HM toxicity; however, it is still in its nascent stages, and further work is necessary to fully address the issue. In conclusion, tackling HM contamination in morels requires ongoing efforts to identify and cultivate low HM accumulation species, use high-quality passivation materials that do not hinder morel growth, and employ a range of strategies to reduce HM toxicity.

## Figures and Tables

**Table 1:** The absorption of HMs by different varieties of morels was reported in some areas.

Morel Varieties	Areas	Heavy Metals (mg kg^−1^)									Reference
		Cd	Cu	Pb	Zn	Hg	As	Mn	Ni	Cr	
*M. esculenta*	Northeastern United States	-	-	2.37	-	-	0.42	-	-	-	[21]
*M. esculenta*	Bulgaria	1.5	18.1	23.0	44.1	-	-	77.5	1.92	1.83	[51]
*M. esculenta*	New Jersey	1.63	-	2.94	101.6	-	-	21.58	-	-	[52]
*M. elata*	Turkey	2.788	18.74	-	95.23	-	-	55.54	9.063	5.468	[53]
*M. deliciosa*	Turkey	1.713	14.17	12.78	100.1	-	-	22.24	5.05	0.408	[53]
*M. steppicola*	Turkey	0.26	15.5	0.28	59.5	0.36	0.61	50.0	13.8	6.6	[54]
*M. esculenta*	Turkey	1.08	42.9	1.43	45	-	-	25.4	1.18	1.05	[55]
*M. conica*	Turkey	0.2	39	1.2	90	0.06	0.25	41	1.1	0.7	[56]
*M. vulgaris*	Turkey	0.89	28	4.2	146	-	-	77	4.0	7.0	[57]
*M. esculenta*	Slovakia	6.169	-	0.291	-	0.052	0.469	-	9.276	1.616	[25]

**Table 2:** Morel heavy metal pollution remediation technologies.

Remediation Process	Techniques	Advantages	Disadvantages	Time-Consuming	Acceptance	Application	Reference
Soil stabilization	Adsorption material/fixatives	Easy preparation, green environmental protection, and waste utilization.	After failure, pollution will reappear and the pollution capacity will increase, so it is not suitable for long-term use.	Medium-term	High	Biochar (Cu, As, Cd, Pb, Hg, Cr); Clay Minerals (Cd, Zn, Cu, Pb); Magnetic Nanoadsorbents (V, Zn, Ni, Cu, Mn); Natural Zeolite (Cd, Co, Cu, Ni, Zn); Silicon-based Additive (Al, As, Cd, Cu, Zn, Cr); Montmorillonite-based Amendments (Cu, Pb, Zn, Cd); Bone Char (Cu, Zn, Pb, Cd).	[106,107,108,109,110,111,112,113]
	Compost	Realize the recycling of waste resources.	Mechanical costs and land costs are high, vulnerable to weather.	Medium-term	Medium	Vermicomposting (As, Cu, Pb, Zn); Spent Mushroom Compost (Fe, Hg, As, Zn, Cd, Cr, Co, Ni, Pb, Cu).	[114,115,116,117]
Microbial remediation	Fungal remediation	The repair effect is good, there are many kinds, low cost, simple operation, green and pollution-free.	Some fungi are small and difficult to separate from the soil. Some fungi compete with Morchella for nutrition and affect their growth.	Medium-term	Low	Galerinavittiformisha (Cu, Cd, Cr, Pb, Zn); *A. niger* (Cd, Cr); *A. Flavus* And *A. niger* (Cu, Pb); Arbuscular Mycorrhizal Fungi (Cd, Cr, Ni, Cu, Pb, Zn); White Rot Fungi (Pb, Cu, Cd, Cr, Ni, Zn, Hg).	[118,119,120,121,122]
	Bacteria remediation	The repair effect is good, there are many kinds, low cost, simple operation, green and pollution-free.	Bacteria are small and difficult to isolate from soil.	Medium-term	Low	*Bacterial mixtures* (Pb, Cu, Cd); Plant Growth Promoting Bacteria (Pb, Cr, Zn, Cd, As, Fe, Cu); *Bacillus* sp., *Lysinibacillus* sp., and *Rhodococcus* sp. (Al, Cd, Cu, Mn, Pb).	[123,124,125]
Variety screening and cultivation	Screening high-quality strains	The operation is simple and only needs to be screened by plate experiment.	It is difficult to collect strains.	Short-term	Low	*Agaricus bisporus* (Pb, Cr, As, Hg, Cd); *Lentinus edodes* (Cd); *Oyster* Mushrooms (Cd).	[126,127,128]
	Radiation breeding	The operation is simple, only the strain is placed near the ray source.	The direction and nature of variation are difficult to predict and control.	Short-term	Low	*Agaricus brasiliensis* (As, Pb, Cd); *Pleurotus ostreatus* (Zn, Fe, Mn, Pb).	[129,130]
	Transgenic breeding	Accurate, direct, and efficient.	The operation is complex, the success rate is low, and it is affected by many factors.	Long-term	Low	*Flammulina velutipes* (Output); *Pleurotus* (Quality); *Pleurotusostreatus* (Zn).	[131,132,133]
	Cross breeding	High success rate, can combine multiple excellent traits.	The breeding process is slow and complex, and trait separation may occur.	Long-term	Low	*Agaricus blazei* (Cd); *Lentinula edodes* (Cd).	[134,135]
Agronomical measures	Multifunction fertilizer	The operation is simple and low-cost.	It may cause secondary pollution.	Short-term	Low	*Pleurotus Tuber-regium* (Fe, Mn, Co, Ni, Zn, Cu, Pb, Cr, Cd, Hg, As); Calcium Magnesium Phosphate Fertilizer (Cu, Zn, Cd, Cr, Pb); Sheep Manure and Potassium Fertilizer (Zn, Cd, Pb).	[136,137,138]
	Rotation/intercropping	It has a wide range of applications and can increase soil organic quality and soil fertility.	The growth is slow and the cycle is long, and the harvest causes secondary pollution.	Long-term	Medium	Peach-Morchella; Morchella Crassipes-Maize; Rice-Vegetables-Morchella.	[15,139,140,141]
	Water management	The method is simple and easy to operate.	It is necessary to measure the content of heavy metals in soil.	Short-term	Low	Mushrooms; *Oryza sativa* (Cd).	[128,142]

“-” indicates not mentioned.

**Table 3:** Comparison of remediation effects of different microorganisms on heavy metals.

Heavy Metal	Bacteria/Fungus	Stress Level (mg kg^−1^)	Efficiency (%)	References
Ni	*Bacillus cereus NS4*	898	95.78%	[176]
	*Sporosarcina globispora (UR53)*	2	89.5	[177]
	*Acidithiobacillus thiooxidans*	13,224.7	48.5	[178]
	*Bacillus* sp., *Paenibacillus* sp.	10	48%	[179]
	*Aspergillus niger*	20.5	41%	[180]
	*Sulfatereducing bacteria*	100	90.1%	[181]
Cd	*Acidithiobacillus thiooxidans*	1.9	93%	[178]
	*Bacillus* sp., *Paenibacillus* sp.	5	56%	[179]
	*Pseudomonaaeruginosa*	25	74.2%	[182]
	*Paecilomyces lilacinus NH1*	0.18	30%	[183]
	*Rhodobacter sphaeroides*	10	67%	[184]
	*Pseudomonas chenduensis*	0.08	30%	[185]
	*Mycobacterium*	15	57.5%	[186]
Cu	*Acidithiobacillus thiooxidans*	41,237.3	53%	[178]
	*Aspergillus niger*	20.82	41.7%	[180]
	*Sulfatereducing bacteria*	100	98.2%	[181]
	*Aspergillus* sp., *Penicillium* sp.	75.11	15%	[187]
Cr	*Sulfatereducing bacteria*	100	99.8%	[181]
	*Exiguobacterium* sp., *Delftia* sp., *Pannonibacter* sp.	9.199	71.08%	[188]
	*Pannonibacter phragmitetus* sp.	462.8	97.8%	[189]
Zn	*Pseudomonaaeruginosa*	25	78.3%	[182]
Hg	*Pseudomonas* sp.	101.5	80%	[190]

## Data Availability

Not applicable.
